# Distribution of invasive versus native whitefly species and their pyrethroid knock-down resistance allele in a context of interspecific hybridization

**DOI:** 10.1038/s41598-022-12373-4

**Published:** 2022-05-19

**Authors:** Alizée Taquet, Hélène Jourdan-Pineau, Christophe Simiand, Martial Grondin, Benoit Barrès, Hélène Delatte

**Affiliations:** 1 Département de Biologie, Université de La Réunion, 15 Avenue René Cassin, CS 92003, 97744 Saint-Denis Cedex 9, La Réunion France; 2 CIRAD, UMR PVBMT, F-97410 Saint-Pierre, La Réunion France; 3 Université de Lyon, Anses, INRAE, USC CASPER, Lyon, France; 4 CIRAD, UMR ASTRE, Campus International de Baillarguet, 34398 Montpellier Cedex 5, France; 5 ASTRE, CIRAD, INRAE, Université de Montpellier, Montpellier, France; 6 CIRAD, UMR PVBMT, Ambatobe, 101 Antananarivo, Madagascar; 7 FOFIFA CENRADERU-DRA, Ambatobe, 101 Antananarivo, Madagascar

**Keywords:** Ecology, Evolution, Genetics, Molecular biology

## Abstract

The invasion success of a species in an agrosystem is greatly influenced by environmental factors such as the use of insecticides, by the intrinsic evolutionary capabilities of the species, and also by interactions with resident species. On the island of La Réunion, the successive invasions of MEAM1 and MED whitefly species over the last 20 years have not only led an increased use of insecticides, but have also challenged the resident IO species. To trace the evolution of the 3 species, and the distribution of the *kdr* mutation (resistance to pyrethroid) in the para-type voltage-gated sodium channel, we genotyped 41 populations (using neutral nuclear markers) and look at the prevalence of the *kdr* allele. MEAM1 was predominantly present in agrosystems showing quasi fixation of the resistant *kdr* allele whereas IO was mainly in natural environments and did not have any resistant allele. Hybridization between the two former species was detected in low frequency but has not led to introgression of resistant alleles in the resident species so far. MED showed a limited distribution in agrosystems but all individuals displayed a resistant allele. These highly contrasting patterns of distribution and resistant mutations between invasive and resident whitefly species are further discussed.

## Introduction

Biological invasions, which could be defined as the successful establishment and spread of species outside their native range^1^, are one of the world’s costliest ecological concerns^2^. They are partly responsible for the loss of biodiversity, by altering ecosystems and natural habitats^3^, disrupting agriculture and posing multiple threats to native species (through competition, predation, hybridization or even introduction of parasites and diseases^4–7^). Not all introduced species settle permanently in a new environment, they must be able to pass through a few critical steps: transport or emigration, introduction, establishment, expansion and proliferation^8,9^. In each of these different steps, evolutionary mechanisms may play an important role^10^.

During biological invasion, previously isolated species or populations can be brought into contact and hybridize. Hybridization can generate new evolutionary solutions, through the pooling of differentiated genetic backgrounds and the increase of genetic variation. Due to the creation of novel genotypes, hybrids may exhibit more extreme transgressive phenotypes (compared to their parents), and may display an enhanced fitness^5,11^. Admixture offers many benefits to invaders, suggesting that hybridization could play an important role in biological invasions^12^. Hybridization can also alter the effectiveness of control strategies toward agricultural pests.

As they arrive in a new environment, invasive species have to face novel selection pressures, biotic as well as abiotic. Invasion success in agroecosystems may be constrained by insecticide treatments. In fact, insecticide use is a major selective force driving the evolution of insect pests and it may also increase the rate of mutations via epigenetic processes^13^. For example, one possible reason for the spread of thrips *Frankliniella* *occidentalis* is that intensive insecticide use in horticulture have selected an insecticide resistant strain, allowing its establishment in glasshouses across North America, and later to Europe, Asia, Africa and Australia^14^. Piiroinen, et al*.*^15^ have demonstrated that the phenotypic traits of insect pests that allow them to thrive under insecticide exposure, may also facilitate global invasions. For instance, sublethal pyrethroid insecticide exposure were found to have transgenerational positive effects on fitness-related traits in the Colorado potato beetle *Leptinotarsa* *decemlineata*^16^. Although many insect pest populations have shown resistance to pyrethroids, it remains one of the most commonly used insecticide class because it shows low risk to mammals^17,18^. A well-known and major pyrethroid resistance mechanism in insects, commonly referred to as knockdown resistance or ‘*kdr*’, involved a mutation on the pyrethroids’ target, the voltage-gate sodium channel gene^19–22^. Thus, monitoring the spread of *kdr* mutant alleles among pest populations in the field is considered to be an accurate method to assess the extent of pyrethroid resistance problems.

*Bemisia* *tabaci* (Gennadius) (Hemiptera: Aleyrodidae) is a highly adaptable insect pest, distributed throughout tropical and subtropical regions worldwide, and responsible for heavy crop losses^23,24^. Because of its ability to transmit more than 200 phytoviruses to an impressive range of host plants, it is threatening food security around the world^25^. *B.* *tabaci* is considered to be a cryptic species complex with at least 40 morphologically indistinguishable species^23,26–28^. Two of these cryptic species are invasive worldwide: the Middle East Asia Minor 1 species (MEAM1) and the Mediterranean species (MED), formerly known as B and Q biotypes, respectively^23,29^.

The MEAM1 whitefly species (originating from the Middle East-Asia Minor region) was responsible of a first worldwide invasion, observed in the late 1980s^24,30^. It has been followed in early 2000s by the MED species, which has spread globally from the countries bordering the Mediterranean basin^23,31^. Both species have evolved resistance to insecticides from most chemical classes^32^, but MED was reported to have a larger spectrum of resistance than MEAM1 to insecticides such as pyriproxyfen and neonicotinoids^33^. This difference between resistance statuses may have played a role in the displacement of the MEAM1 species by the MED species in some countries^34^.

In the island of La Réunion, three species of this whitefly complex have been described. The indigenous species Indian Ocean (IO), coexisting with two invasive species: MEAM1 and MED. The arrival of the MEAM1 species was dated to the late 90’s, together with the first description of the *Tomato* *yellow* *leaf* *curl* *virus* (TYLCV, genus: *Begomovirus*, family: *Geminiviridae*)^35,36^, that implied high yield losses on tomato crops, and the use of phytosanitary control measures for this pest^37^. Two studies based on the analysis of the genetic diversity of the whitefly populations have described the occurrence of interspecific hybridization between both MEAM1 and IO species^38,39^. Then in 2010, the MED species was first detected on the island^40^. Therefore, to further explore the interactions between those resident and invasive species in agrosystems, subjected to high insecticide pressure, we studied (i) the evolution of the distribution of the three species since their first description on the island, (ii) their genetic diversity and structure, and (iii) the evolution of the interspecific hybridization, in relation to the distribution of the *kdr* alleles conferring resistance to pyrethroids.

## Results

### Species identification and distribution

**Figure 1:**
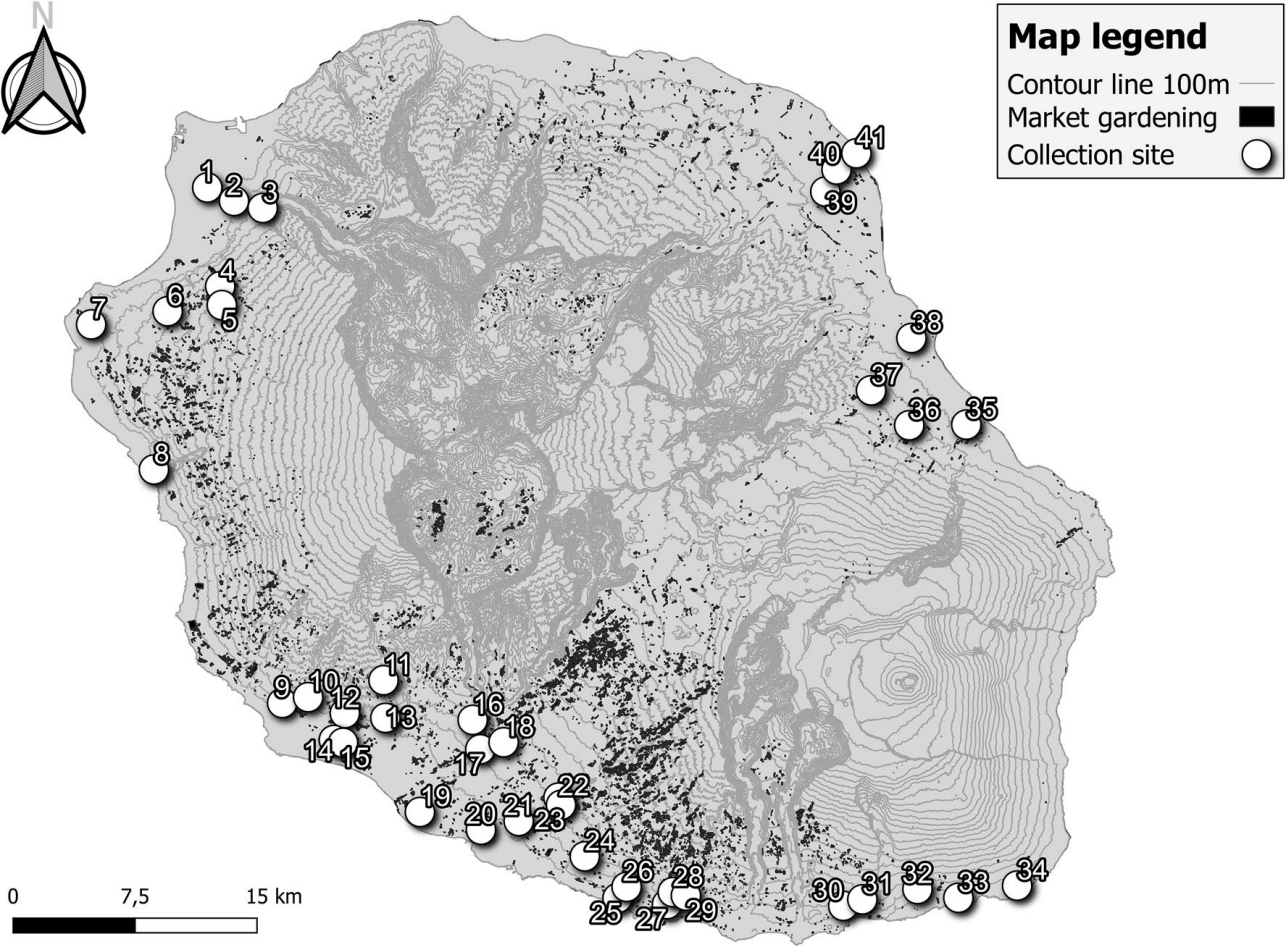
Global repartition of *B.* *tabaci* collection sites in and around market gardening areas of La Réunion. Each site is named as referred to in Table 1.

**Figure 2:**
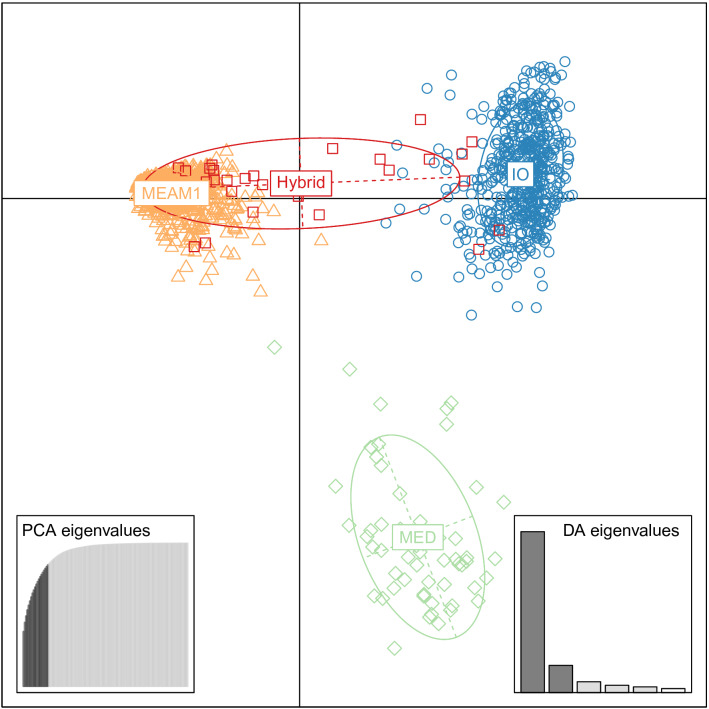
DAPC based on n = 1562 individuals using 11 microsatellite loci. Dots of different colours indicate individuals from different genetic clusters. PCs eigenvalues and discriminant factors retained are indicated.

1562 adult whiteflies sampled in 41 different sites around the island were genotyped (Fig. 1). Bayesian clustering analyses performed with STRUCTURE and the DAPC allowed the clear determination of three main genetic groups in our dataset (Fig. 2). The sequencing of the COI barcoding region of 286 individuals from each group identified these groups to the three species already present in La Réunion: MEAM1, IO and MED. All sequences had 100% identity with accessions already published on La Réunion: no. AJ550175 for MEAM1, no. AJ877264 for IO, no. JN090173 for MED (Q1).

The whole dataset comprises 61.1% of MEAM1, 33.5% of IO and 3.5% of MED, respectively (Table 1). In the sampled sites, MEAM1 was mainly found in greenhouses, open fields and field surroundings. The IO species was found mostly in non-cultivated areas and in field surroundings. Finally, the MED species was a minority present in 4 sites, only in the cultivated areas of the south-west of La Réunion.

**Table 1:** *B.* *tabaci* sampling in La Réunion: location of collection site, environment, host plant and number (N) of species and hybrids found.

Site	GPS coordinates			Environment	Host plant	N of *B.* *tabaci*			
	Latitude	Longitude				MEAM1	IO	Hybrid	MED
1	20°57′55.7′′S	55°18′06.2′′E		Open field	Tomato	29	1	1	0
2	20°58′23.1′′S	55°19′02.8′′E		Field surroundings	Weeds	1	29	1	0
				Greenhouse	Tomato	3	25	1	0
3	20°58′37.78′′S	55°20′3.75′′E		Non-cultivated	Weeds	0	3	1	0
4	21°1′13.58′′S	55°18′31.53′′E		Greenhouse	Tomato	30	0	0	0
5	21°1′51.26′′S	55°18′34.51′′E		Field surroundings	Weeds	0	12	0	0
6	21°2′2.35′′S	55°16′39.00′′E		Non-cultivated	Weeds	0	32	0	0
7	21°2′27.08′′S	55°13′55.82′′E		Field surroundings	Weeds	1	19	5	0
				Open field	Tomato	4	22	1	0
8	21°7′18.71′′S	55°16′6.19′′E		Non-cultivated	Weeds	4	27	1	0
9	21°15′8.68′′S	55°20′34.19′′E		Open field	Tomato	12	0	0	9
10	21°14′57.55′′S	55°21′29.75′′E		Open field	Tomato	2	27	0	0
11	21°14′25.14′′S	55°24′10.97′′E		Field surroundings	Weeds	12	0	0	0
				Open field	Tomato	14	2	0	1
12	21°15′30.45′′S	55°22′47.66′′E		Greenhouse	Tomato	26	0	0	0
13	21°15′40.57′′S	55°24′14.12′′E		Field surroundings	Weeds	22	2	0	0
14	21°16′23.61′′S	55°22′23.62′′E		Non-cultivated	Weeds	3	19	1	0
15	21°16′29.39′′S	55°22′43.24′′E		Field surroundings	Weeds	18	0	1	0
				Open field	Tom + egg^a^	52	1	0	2
16	21°15′46.3′′S	55°27′20.3′′E		Non-cultivated	Weeds	4	28	0	0
17	21°16′45.15′′S	55°27′35.90′′E		Field surroundings	Weeds	28	0	0	1
				Greenhouse	Tomato	15	0	0	17
				Open field	Eggplant	38	0	1	24
18	21°16′31.74′′S	55°28′25.77′′E		Field surroundings	Weeds	32	0	0	0
19	21°18′49.62′′S	55°25′25.70′′E		Field surroundings	Weeds	30	0	0	0
				Greenhouse	Tomato	31	0	1	0
20	21°19′26.19′′S	55°27′35.63′′E		Field surroundings	Weeds	5	15	0	0
				Greenhouse	Cucumber	29	0	1	0
21	21°19′9.59′′S	55°28′56.36′′E		Open field	Eggplant	25	1	5	0
22	21°18′23.47′′S	55°30′21.69′′E		Field surroundings	Weeds	21	0	0	0
				Greenhouse	Melon	26	0	0	0
23	21°18′38.17′′S	55°30′27.02′′E		Field surroundings	Weeds	11	3	0	0
				Greenhouse	Eggplant	42	0	0	0
24	21°20′21.56′′S	55°31′16.66′′E		Field surroundings	Weeds	20	3	1	0
				Greenhouse	Tomato	28	0	0	0
25	21°21′45.58′′S	55°32′25.80′′E		Greenhouse	Melon	59	0	0	0
26	21°21′23.31′′S	55°32′45.36′′E		Greenhouse	Tomato	27	0	0	0
				Field surroundings	Weeds	2	28	1	0
27	21°21′58.64′′S	55°34′11.30′′E		Greenhouse	Melon	30	0	2	0
28	21°21′34.59′′S	55°34′23.86′′E		Greenhouse	Tomato	2	0	0	0
29	21°21′41.91′′S	55°34′51.80′′E		Non-cultivated^b^	Weeds	2	30	0	0
30	21°22′5.20′′S	55°40′27.56′′E		Open field	Tomato	32	0	0	0
31	21°21′52.63′′S	55°41′8.43′′E		Greenhouse	Tomato	31	0	0	0
32	21°21′32.80′′S	55°43′6.28′′E		Greenhouse	Tomato	31	0	1	0
33	21°21′50.95′′S	55°44′33.44′′E		Non-cultivated	Weeds	0	26	0	0
34	21°21′27.50′′S	55°46′39.13′′E		Greenhouse	Eggplant	27	0	1	0
35	21°6′4.21′′S	55°44′58.39′′E		Non-cultivated	Weeds	0	22	0	0
36	21°6′4.55′′S	55°42′56.75′′E		Field surroundings	Weeds	0	25	1	0
37	21°4′54.13′′S	55°41′36.01′′E		Non-cultivated	Weeds	0	10	0	0
38	21°3′9.99′′S	55°43′2.98′′E		Non-cultivated	Weeds	0	23	0	0
39	20°58′15.49′′S	55°40′2.19′′E		Field surroundings	Weeds	30	1	0	0
				Open field	Eggplant	32	0	0	0
				Greenhouse	Tomato	32	0	0	0
40	20°57′31.51′′S	55°40′26.43′′E		Field surroundings	Weeds	0	23	0	0
				Open field	Tomato	0	34	1	0
41	20°56′59.50′′S	55°41′8.85′′E		Non-cultivated	Weeds	0	31	0	0
Total N of *B.* *tabaci* species and hybrids						955	524	29	54

Weeds: Mexican fireplant *Euphorbia* *heterophylla* L. (Euphorbiaceae), bean *Vigna* *sp.* L. (Fabaceae), lantana *Lantana* *camara* L. (Verbenaceae), pricklyburr *Datura* *innoxia* Mill. (Solanaceae), turpeth *Operculina* *turpethum* (Convolvulaceae), cotton *Gossypium* *sp.* (Malvaceae); crops: tomato *Solanum* *lycopersicum* L. (Solanaceae), eggplant *Solanum* *melongena* L. (Solanaceae), cucumber *cucumis* *sativus* L. (Cucurbitaceae), melon *Cucumis* *melo* L. (Cucurbitaceae).

*NA* not available.

^a^Host plants are tomato and eggplant crops.

^b^Sugarcane field without any insecticide treatment.

### Genetic diversity

No linkage disequilibrium was observed between all pairs of loci tested within each species, and between each pair of populations of those species. The average allelic richness was very similar between the three species, with in average 2.4 ± 0.03 (SE) for MEAM1, 2.5 ± 0.03 for IO and 2.3 ± 0.06 for MED, respectively. A higher level of heterozygosity was observed for IO (in average over all populations: 40.3% ± 0.7%) than for the two other species (29.7% ± 0.6% for MEAM1 and 30.3% ± 3.3% for MED). The F_IS_ values ranged from -0.05 to 0.25 for MEAM1, from -0.76 to 0.096 for IO and 0.005 to 0.354 for MED, respectively. Significant departures from Hardy–Weinberg equilibrium were observed for 12 populations (out of 34) of the MEAM1, for 4 populations (out of 21) of the IO, and for 2 populations (out of 3) of the MED (Table 2).

**Table 2:** Population genetic diversity indices.

Species	Site	Environment	N of *B.* *tabaci*	*Na*	*Ra*	*Ho*	*He*	*Fis*
MEAM1	1	Open field	29	43	2.541	0.299	0.324	0.096
	4	Greenhouse	30	40	2.386	0.267	0.323	0.190***
	9	Open field	12	36	2.548	0.356	0.344	0.010
	11	Field surroundings	12	28	2.155	0.235	0.283	0.213
	11	Open field	14	30	2.199	0.260	0.292	0.146
	12	Greenhouse	26	42	2.396	0.357	0.344	−0.023
	13	Field surroundings	22	44	2.628	0.295	0.364	0.222**
	15	Field surroundings	18	42	2.429	0.299	0.318	0.087
	15	Open field	52	47	2.473	0.330	0.348	0.061***
	17	Field surroundings	28	41	2.436	0.308	0.330	0.085*
	17	Greenhouse	15	38	2.544	0.351	0.347	0.024
	17	Open field	38	42	2.464	0.287	0.330	0.143
	18	Field surroundings	32	38	2.073	0.270	0.263	−0.011
	19	Field surroundings	30	40	2.274	0.285	0.303	0.077
	19	Greenhouse	31	40	2.335	0.295	0.311	0.067*
	20	Field surroundings	5	21	1.909	0.236	0.264	0.212
	20	Greenhouse	29	44	2.426	0.325	0.328	0.028
	21	Open field	25	46	2.551	0.364	0.363	0.019
	22	Field surroundings	21	35	2.254	0.282	0.316	0.131
	22	Greenhouse	26	45	2.489	0.259	0.338	0.250***
	23	Field surroundings	11	27	2.184	0.273	0.311	0.169
	23	Greenhouse	42	46	2.389	0.292	0.323	0.107*
	24	Field surroundings	20	37	2.361	0.330	0.311	−0.026
	24	Greenhouse	28	50	2.445	0.291	0.332	0.143
	25	Greenhouse	59	44	2.347	0.296	0.323	0.093**
	26	Greenhouse	27	42	2.370	0.283	0.317	0.125**
	27	Greenhouse	30	44	2.364	0.261	0.312	0.173**
	30	Open field	32	45	2.500	0.318	0.331	0.054*
	31	Greenhouse	31	37	2.456	0.328	0.339	0.049
	32	Greenhouse	31	38	2.130	0.238	0.272	0.142
	34	Greenhouse	27	38	2.362	0.300	0.338	0.131***
	39	Field surroundings	30	42	2.395	0.288	0.330	0.145
	39	Open field	32	44	2.343	0.330	0.309	−0.052
	39	Greenhouse	32	39	2.300	0.295	0.308	0.056
IO	2	Field surroundings	29	38	2.511	0.386	0.392	0.034
	2	Greenhouse	25	35	2.579	0.392	0.391	0.022
	5	Field surroundings	12	28	2.461	0.420	0.400	−0.009
	6	Non-cultivated	32	33	2.382	0.414	0.394	−0.037***
	7	Field surroundings	19	31	2.525	0.422	0.414	0.013
	7	Open field	22	31	2.497	0.382	0.387	0.037
	8	Non-cultivated	27	32	2.377	0.417	0.386	−0.061
	10	Open field	27	40	2.834	0.430	0.430	0.018**
	14	Non-cultivated	19	31	2.511	0.390	0.401	0.058
	16	Non-cultivated	28	28	2.233	0.344	0.353	0.043
	20	Field surroundings	15	29	2.447	0.335	0.352	0.084
	26	Field surroundings	28	35	2.655	0.408	0.392	−0.021
	29	Non-cultivated	30	31	2.255	0.371	0.369	0.011
	33	Non-cultivated	26	31	2.421	0.461	0.414	−0.092
	35	Non-cultivated	22	31	2.419	0.407	0.392	−0.011
	36	Field surroundings	25	35	2.516	0.420	0.392	−0.049
	37	Non-cultivated	10	28	2.507	0.386	0.382	0.044
	38	Non-cultivated	23	36	2.684	0.452	0.414	−0.066*
	40	Field surroundings	23	32	2.392	0.419	0.382	−0.076
	40	Open field	34	35	2.667	0.390	0.424	0.096*
	41	Non-cultivated	31	35	2.583	0.415	0.407	−0.003
MED	9	Open field	9	30	2.363	0.240	0.330	0.354
	17	Greenhouse	17	35	2.218	0.316	0.308	0.005*
	17	Open field	24	40	2.445	0.353	0.375	0.087*

Species of whiteflies, site number where individuals were collected (as referred to in Table 1), number of individual genotyped (N), mean number of alleles per population (Na), allelic richness (Ra), observed heterozygosity (Ho), expected heterozygosity (He), and fixation indices (Fis) presented together with Bonferroni corrected *p* value from Hardy–Weinberg equilibrium test (*** ≤ 0.001/m < ** ≤ 0.01/m < * ≤ 0.05/m, m being the number of populations tested for each species. m = 34, 21 and 3 for MEAM1, IO and MED, respectively), are indicated.

The analysis of bottleneck performed with the SMM model did not reveal any significant signal on IO populations, whereas almost all MEAM1 populations had undergone a significant bottleneck (P < 0.05). On the contrary, the IAM model indicated no bottleneck for both species.

### Population structure

Within species, the genetic differentiation between populations was low (overall F_ST_ for IO = 0.007, MEAM1 F_ST_ = 0.03, and MED F_ST_ = 0.07). In MEAM1, no significant genetic differentiation was found between different environments (greenhouse, open field or field surroundings, see Table 1) within sites; however, several pairwise genetic differentiations between distant sites were significant (see Supplementary Table S1 online). Nevertheless, no substructure was observed between groups of individuals of this species. No indication of isolation by distance for the MEAM1 species was found (Mantel tests *p*-value > 0.05).

Similar results were observed for IO populations, showing no substructure between groups of individuals or looking at F_ST_ genetic distances between different environments (see Supplementary Table S2 online). Indeed, only a single pairwise genetic distance was found to be significantly different (site 16 with site 41, two sites that were far apart: east and west coast of the island, respectively; Table 1, Fig. 1).

**Figure 3:**
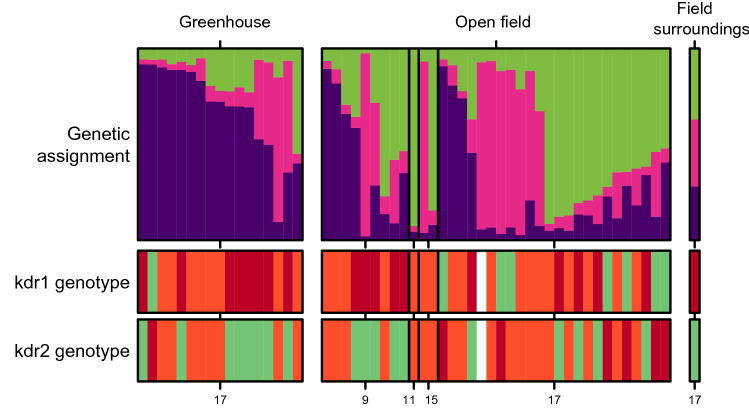
Genetic assignment (Bayesian assignment to K = 3 clusters, purple, pink and green bars) and kdr genotype at the loci L1 and L2 (green for SS, orange for RS and red for RR) for MED individuals sampled in greenhouse, open field and field surroundings. Missing data are indicated by white bars. Numbers on the X-axis (9, 11, 15 and 17) correspond to sampling sites as referred to in Table 1.

The MED species was the only species for which a substructure was observed with the Bayesian analysis (Fig. 3), with the best number of clusters of three. However, those clusters did not reflect differentiation between sampled sites or other obvious environmental constraints. It has to be noticed that this result involves a very small number of samples (n = 50), spread over four sites, close to each other (Fig. 1).

### Hybrids

**Figure 4:**
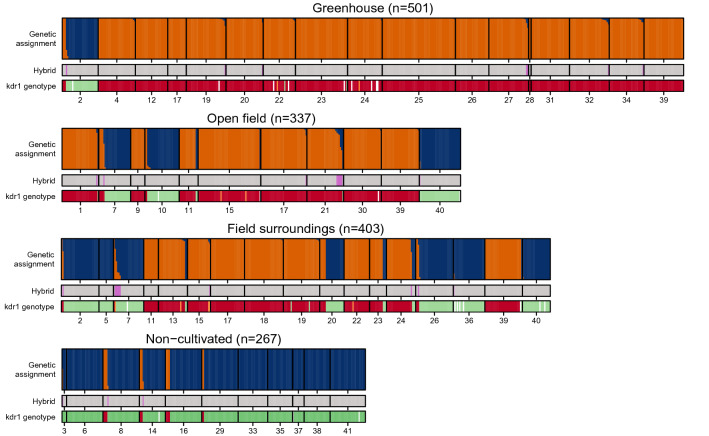
Genetic assignment to the MEAM1 (orange) and the IO (blue) genetic clusters, identification of hybrids (pink bars) and kdr genotype at the L1 locus (green for SS, orange for RS and red for RR) of individuals sampled in greenhouse, open field and field surroundings. Missing data are indicated by white bars. Individuals are ordered by their probability of assignment to the clusters. Numbers on the X-axis correspond to sampling sites as referred to in Table 1.

Individuals were considered hybrid when their Bayesian assignment (Fig. 4) to MEAM1 or IO clusters was between 10 and 90%, they were also well separated on the X-axis of the DAPC analysis (Fig. 2). Accordingly, there were 29 hybrids, which represents only 1.9% of the whole dataset. We found as many interspecific mating with female IO or with female MEAM1 (13 versus 16). The distribution of assignment probabilities indicate that most hybrids derived from backcrosses, mostly toward the MEAM1 species. Hybrids were found in all environments. However, assignment probabilities of hybrids were significantly linked with the sampled environment: individuals derived from backcrosses with MEAM1 (respectively IO) were preferentially found in agrosystems (respectively non-cultivated areas; χ^2^ = 8.28, *p*-value = 0.040) (see Supplementary Fig. S1 online).

### *Kdr* mutation

**Figure 5:**
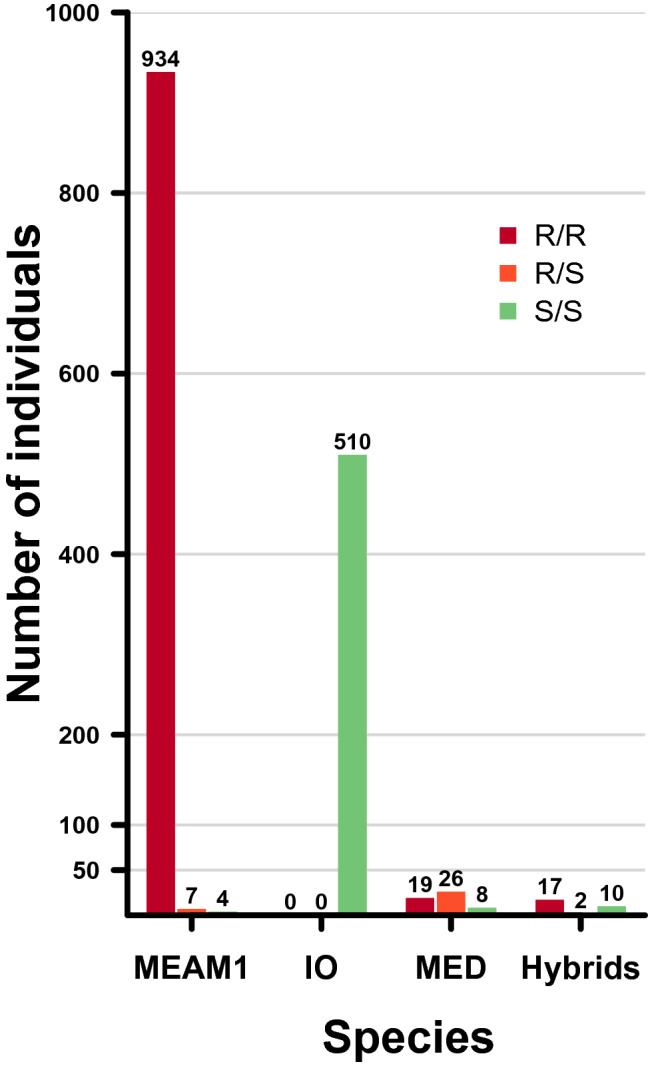
Resistance genotypes of *B.* *tabaci* species collected in La Réunion, at a point mutation in the IIS4-5 linker of para-type voltage gated sodium channel gene: L925I. I925 and L925 are the resistant (R) and the susceptible (S) alleles, respectively.

The region of the sodium channel gene carrying the *kdr* mutation was successfully genotyped for 98% (n = 1537) of the sampled individuals (Fig. 5). The L925I resistance mutation (here referred to as L1) was detected in different frequencies within species: 0.99 for MEAM1, 0.62 for MEAM1-IO hybrids, 0.60 for MED and 0 for IO.

Individuals of the MEAM1 species were overwhelmingly (99%) homozygous resistant (RR), whereas all individuals of the IO species were homozygous susceptible (SS) at this locus (Figs. 3, 5). Those results were confirmed by sequencing the *kdr* region for a fraction of individuals (L1, Table 3).

**Table 3:** Resistance genotype frequency of the sodium channel gene for MEAM1, IO, MEAM1-IO hybrids and MED DNA samples collected in La Réunion.

Species	N	Genotypes		
		L1	L2	L3
MEAM1 (N = 58)	49	A/A	AC/AC	T/T
	4	A/T	AC/AC	C/T
	1	A/T	AC/GT	C/T
	4	T/T	AC/AC	C/C
IO (N = 34)	34	T/T	AC/AC	C/C
Hybrids (N = 28)	17	A/A	AC/AC	T/T
	1	A/T	AC/AC	C/T
	10	T/T	AC/AC	C/C
MED (N = 50)	16	A/A	AC/AC	C/C
	26	A/T	AC/GT	C/C
	1	T/T	AC/GT	C/C
	7	T/T	GT/GT	C/C

Three loci were investigated: L1 (L925I), L2 (T929V) and L3 (the latter being in the intron region). I925 (‘A’ at L1) and V929 (‘GT’ at L2) are the resistant alleles, whereas L925 (‘T’ at L1) and T929 (‘AC’ at L2) are the susceptible alleles.

MED individuals showed a combination of RR (~ 36%), RS (~ 49%) and SS (~ 15%) at this L1 locus (Figs. 3, 5), and displayed a mutation conferring resistance to pyrethroids at a second locus (T929V; frequency = 0.41), here referred to as L2^41^ (Table 3). All MED individuals sequenced carried at least one resistance allele (Fig. 3). This mutation at the L2 locus was also observed in one MEAM1 individual (frequency = 0.01; n = 58), but was totally absent in IO as in MEAM1-IO hybrids (Table 3).

Another mutation was detected in the intron region right after the 5’ end of all sequences of IO and MED species (locus L3, Table 3). In MEAM1, only 9 over the 58 sequenced individuals carried this mutation among which 5 were heterozygous both at this locus and at the L1 locus and 4 were homozygous at this locus and SS at the L1 locus.

For the hybrids, the *kdr* genotype was linked with assignment probability to parental species (χ^2^ = 18.616, *p* value = 9.069e^−05^). Hybrid individuals backcrossed to IO were all homozygous susceptible (SS), whereas two individuals backcrossed towards MEAM1 were heterozygotes (RS). The mutation at the L3 locus was also detected in 11 out of the 29 hybrids (Table 3), most of which (9/11) were backcrossed towards IO.

To summarize, if we consider both resistance mutations (L1 and L2) when analyzing the genotyping results presented in Fig. 5 and Table 3: IO appeared 100% susceptible, as we did not detect any resistance allele at any locus; MED appeared 100% resistant, as each individual carried at least one resistance allele at one locus (L1, L2 or both). For MEAM1, we detected 99% of resistant individuals, as they carried at least one resistance mutation at one locus (L1, L2 or both). Concerning MEAM1-IO hybrids, 65% of individuals seemed to be resistant as they carried at least one resistance mutation (at L1).

## Discussion

The *B.* *tabaci* complex species in La Réunion is still composed of three species: MEAM1, IO and MED. The three species, which coexist on this relatively small island, display contrasting patterns in terms of ecological niches occupancy, genetic structure and diversity, and resistant genotypes in response to anthropic pressures.

Our study clearly indicates that the resident indigenous species IO was mainly associated with non-cultivated environments or field surroundings, whereas the two invasive species were almost restricted to agrosystems. Those ecological preferences were expected according to previous studies conducted in La Réunion, where MEAM1 was dominant on vegetable crops whereas IO was found on weeds^38,40,42^. In addition to host plant preferences, non-agricultural environments or wild environments are more likely to be non-pesticide environments.

MEAM1 species, an invader of this insular ecosystem, was first reported in 1997 in the southern part of the island, and in a very short period of time (3 years) it has colonized the entire tomato growing area of the west coast of the island. In 2003, MEAM1 was found in the whole fringe around the island (0–600 m above sea level), including the eastern part. Nevertheless, despite its resident status of over 20 years on this island, it seems that this species has not yet reached an equilibrium state, with lower heterozygosity compared to the indigenous species, genetic differentiation between distant sites (but without any IBD), and still detection of signals of bottlenecks. However, the absence of nuclear substructure within MEAM1 species, moderate genetic diversity and low COI haplotype diversity (no new haplotype found), are not in favor of a new recent invasion or multiple invasions of this species on the island, but might be the signal of a founder effect on the initial invasive population. On the contrary, IO was found to have a higher level of heterozygosity and higher diversity compared to both invasive species, with most of its sampled populations being at the equilibrium of Hardy–Weinberg. In addition, no bottleneck was found associated to any populations of this species, and low levels of genetic differentiation even between distant populations are in agreement with an indigenous, well-established species.

Opposite to the evolution of MEAM1, there is no demographic or geographic expansion of the worldwide invasive MED species. MED was first detected in 2010 in La Réunion^40^, on eggplant in Saint-Pierre (south part of the island). Since then, this species has only been found in four very close localities of the southern part of the island (mostly on crops), less than 30 km from the epicenter of the invasion. MED is expected to have higher competitive abilities compared to MEAM1, as indicated by the frequent replacement of MEAM1 by MED observed in several countries: in China, Japan, South Korea or Spain^31,43–47^. This replacement can even be fairly rapid as in China or in Florida, MED replaced MEAM1 in less than 5 years^48,49^. It was hypothesized that the higher competitive ability of MED compared to MEAM1 was linked to a higher insecticide resistance in the former species^33,46,50^. It appears that MED has not expanded its range during the last 8 years in La Réunion, and did not displaced MEAM1 in agricultural areas. Genotyping and sequencing of the *kdr* mutation indicates that the MED species in La Réunion is as well armed against pyrethroids as MEAM1, but this species may lack other resistance mechanisms in those particular populations. Unfortunately, because of the low occurrence of the MED species in La Réunion, insecticide resistance could not be assessed using bioassays so far^51^.

Finally, we found a genetic structure within the MED species. This substructure, which does not match with any factors explored here, might also reflect a potential signal of multiple invasion events for this species, as suggested in early studies using either microsatellites markers^40^, or SNP data over the whole genome^52^. However, the limited number of MED individuals sampled prevents from further analyses on this aspect.

We found around 2% of hybrids between MEAM1 and IO, the two major species found in La Réunion, in our dataset. This confirms that the reproductive isolation between the two species is incomplete^38,53^. A few individuals were found to be first generation hybrids, but most were found to result from multiple generations of backcrossing between the two species. Hybrids from backcrosses were frequently assigned more strongly to the MEAM1 species, which may be explained by the predominance of this species in the sampled sites. Hybrids between those two species have been described in La Réunion since 2006. In the early invasion process of MEAM1 (2001–2002) on the island, higher rate of hybridization was found, reaching up to 38%^38^, then it decreased over years reaching around 11% in 2006^53^. This decrease in the frequency of hybrids may either be due to the sampled environments: a larger diversity of environments was studied in 2001–2006 in both studies. Another hypothesis could be that the MEAM1 species is progressively genetically overwhelming the IO species in the agricultural niche, because of its greater fitness in this environment. In La Réunion, these two species have been shown recently to radically differ in terms of insecticide resistance, the MEAM1 species being highly resistant to two heavily used insecticides, whereas the IO species seems to remain susceptible to both insecticides^51^.

All the MED individuals in La Réunion displayed either one or the two mutations conferring the *kdr* resistance to pyrethroids, with 26 individuals carrying both mutations. Such a high level of allele frequencies has never been reported, at least never to that extent^54^. This result might be explained by (i) a low number of samples; (ii) the fact that MED populations were found in agrosystems and might have been selected due to insecticide pressure. In contrast, the second mutation (L2) was never found in our sampled MEAM1 individuals as expected^55^, except from one individual. Further investigation should be conducted to assess the occurrence of this mutation (hybridization between both species, high selection pressure on those populations leading to this mutation…).

Our study also reveals a contrasted pattern in the frequency of the first *kdr* mutation (L1) between the invasive MEAM1 and the resident IO species. The L1 mutation has been found to be nearly fixed in La Réunion MEAM1 populations, and fully absent from the IO populations. In Cyprus, several MEAM1 populations displayed high frequency of this allele and also showed an association between the frequency of the resistant allele and bifenthrin (a pyrethroid insecticide) resistance^56^. We have not formally tested the association between genotypic and phenotypic resistance to pyrethroids. However, we estimated the LC_50_ associated with deltamethrin for several MEAM1 populations and found that it was very high, which is in agreement with the high frequency of resistant allele (> 150 mg.L^−1^ of Decis Protech^®^). Recently, Gnankiné, et al*.*^57^ showed that in less than 10 years this mutation (initially present in 2% of the population) reached complete fixation for all MED (MED Q and MED ASL) populations in Burkina Faso. Such a surge in a resistance allele frequency would not take place without a high selection pressure linked to a heavy use of pyrethroid insecticides. We do not know if the same phenomenon occurred for MEAM1 in La Réunion where pyrethroid are commonly used, but it can be hypothesized. Another explanation would be that the mutation was already fixed in the MEAM1 individuals which invaded La Réunion in the late 90’s. Indeed, resistance to pyrethroids was described in whiteflies as early as 1995^58^.

In contrast to MEAM1, the *kdr* mutation was never found in the IO populations, which implies either that it was not subjected to the same insecticide pressure as MEAM1 (i.e., IO is mostly found out of agrosystems^38,42^), or that it was not able to evolve resistance due to molecular constraints^59^. We hypothesized that the hybridization between MEAM1 and IO could have allowed the introgression of resistant alleles from the invasive species to the indigenous one. However, we found no indication of introgression of any *kdr* allele from MEAM1 to IO, but rather a potential introgression of the susceptible allele from IO to MEAM1. Furthermore, the mutation (L3) found in the exon, always associated with IO genotypes and in a few hybrid genotypes, can also be considered as an additional marker of introgression from IO to MEAM1. The susceptible allele is present at low frequency in the MEAM1 species. This further suggests that the initial invasive MEAM1 populations came with the resistant allele in the homozygous state, whereas this susceptible allele might originate from the IO species. Thus, the hybridization between MEAM1 and IO could explain the maintenance of a low frequency of susceptible alleles in MEAM1, despite a probable strong counter-selection of these alleles in the homozygous state. The level of dominance of *kdr* alleles is not known in *B.* *tabaci*, but a codominance between resistant and susceptible alleles has been described in other system, for example in a Lepidoptera species^60^. Heterozygotes would therefore have a better fitness than susceptible homozygotes when treated with insecticide, and would thus be less counter-selected.

The lack of introgression of *kdr* alleles in the other direction from MEAM1 to IO may seem surprising. One hypothesis would be that this resistant allele has a higher fitness cost for IO than for MEAM1. This could be explained if MEAM1 has a reduced cost of resistance according to the selection of a modifier gene elsewhere in the genome. In this case, IO needs to acquire both the resistant allele and the modifier gene to avoid counter-selection in absence of insecticide treatment. This would decrease the probability of introgression of the resistant allele in IO genome. The low number of hybrids leads to caution regarding the conclusions that can be drawn, however, we did not find any indication of a role of hybridization in the evolution of resistance in whitefly in La Réunion.

Following the two invasion events, the whitefly species distribution has not drastically evolved in the past years, and each species seems to be contained in its initial ecological niche. The initial hypothesis of indigenous species not being equally armed regarding to insecticide resistance, facing both invasive MEAM1 and MED species, is validated. The potential insecticide pressure of MED and MEAM1 can be analyzed and discussed in conjunction with more detailed local pesticide practices. Very high levels of resistance for both invasive species were found together with generally high gene flow between whitefly populations of the broadly spread MEAM1 species. As a conclusion, the non-spread of the invasive MED in the insular environment cannot only be explained by differential levels of pyrethroid resistance, but other insecticide resistance or factors must be in play, in particular ecological or environmental factors.

## Methods

### *Bemisia tabaci* collection and DNA extraction

La Réunion is a French subtropical island, with a high-altitudinal gradient (from 0 to 3071 m asl), located in the southwest Indian Ocean at 700 km east of Madagascar. Adult whiteflies were sampled in 41 different sites of this island, from April 2016 to December 2017 (Table 1 and Fig. 1). Sampling was mostly done in agricultural areas, all over the island, along the coastal strip from 0 to 500 m asl, where market gardening production is more important. In some sites, different environments were sampled: greenhouse, open field, field surroundings or non-cultivated area.

At each collection site, GPS coordinates were recorded and about 50 individuals were sampled at random on crops or weeds, by vacuuming plant foliage with a mouth aspirator. According to the research method, it was not possible to determine whether there is mixed population on a single plant. Cultivated host plants included tomato *Solanum* *lycopersicum* L. (Solanaceae), eggplant *Solanum* *melongena* L. (Solanaceae), cucumber *Cucumis* *sativus* L. (Cucurbitaceae), and melon *Cucumis* *melo* L. (Cucurbitaceae); weeds included Mexican fireplant *Euphorbia* *heterophylla* L. (Euphorbiaceae), bean *Vigna* *sp.* L. (Fabaceae), lantana *Lantana* *camara* L. (Verbenaceae), pricklyburr *Datura* *innoxia* Mill. (Solanaceae), and turpeth *Operculina* *turpethum* L. (Convolvulaceae). The study complies with local and national guidelines on field studies on cultivated and wild plants.

Adult whiteflies were conserved in tubes containing 95% ethanol, and held at −20 °C in the laboratory until DNA extraction. Collected adult whiteflies were sexed under a Leica MZ6 stereomicroscope^61^. Indeed, only adult females were used for the population genetic analyses due to the haplo-diploid status of the species. A non-destructive DNA extraction method was used as described in Tocko-Marabena, et al*.*^62^. Then, extractions were conserved at −30 °C until further use.

### Mitochondrial DNA amplification and sequencing

Taxonomic identification of the cryptic species of the *B.* *tabaci* complex is based on the sequencing of the partial 3′ mitochondrial cytochrome oxidase I gene (COI)^23^. Polymerase chain reactions (PCR) were performed as described in Ally, et al*.*^63^, using the primer pair designed by Mugerwa, et al*.*^26^ (see Supplementary Table S3 online). DNA Amplicons were then sent to Macrogen Europe laboratory for sequencing. Sequences were manually edited and aligned using Geneious™ software R10.2.6^64^, and then compared with reference sequences from GenBank using the BLAST algorithm.

### Nuclear microsatellite PCR amplification and genotyping

Nuclear microsatellite PCR amplification was done using 11 loci, combined in three multiplex primer reactions (see Supplementary Table S3 online). PCR amplification, dilution of PCR products, formamide denaturation of DNA and genotyping was according to Ally et al.^63^. Nuclear microsatellite genotyping was carried out with an Applied Biosystem© 3130XL DNA sequencer. Peaks were scored manually using GeneMapper™ software v4.0 (Applied Biosystems, Foster City, CA).

### Population genetic analysis

Micro-checker software was used to correct genotyping errors in our microsatellite data, identifying non-amplified alleles, short allele dominance and the scoring of stuttering peaks^65^. For each of our three cryptic species, mean number of allele per population, allelic richness, observed heterozygosity, expected heterozygosity, and fixation indices^66^ were calculated using Genepop v4.7^67^. Hardy–Weinberg equilibrium was tested and *p*-value adjusted with Bonferroni correction. Linkage disequilibrium was tested with Genepop between pairwise populations and markers. The software Bottelneck v1.2.02^68^ was used to test the temporary excess of heterozygosity that results from a decrease of the effective population size. Deviations from expected heterozygosity were computed through 1,000 permutations, with the stepwise mutation model (SMM) and the two-phased model of mutation (TPM).

### Population structure analysis

Bayesian clustering analyses were performed to assess genetic population structure between our populations. The first analysis was done with Structure software (2.3.4 version)^69^. We first analyzed the whole dataset, including MEAM1, IO and MED species; setting Structure with a 100,000 burn in iteration (10%) and followed by 1,000,000 Markov Chain Monte-Carlo (MCMC) iterations, using an admixture model allowing correlation between allele frequencies between populations. The number of assumed clusters (K) was set to a range going from 1 to 20, each step being repeated 5 times. The best K was estimated by means of *∆k* as described by Evanno, et al*.*^70^, using the online program Structure Harvester^71^. Then, a Discriminant Analysis of Principal Components (DAPC) was performed in R^72^, between those genetic clusters, using the ‘adegenet’ package v2.1.3^73^. The two (Structure and DAPC) analyses were congruent and analyses were further carried out.

Because of the existence of hybrids between both MEAM1 and IO species^38^, we analyzed a first subset excluding MED species, in order to better identify these individuals. We further split the whole dataset into three other subsets, each species being taken alone to decipher their substructure. For these four analyses of population structure (MEAM1 & IO, MEAM1, IO and MED), Structure was also set with 100,000 burn-in length with run length of 1,000,000 MCMC. The number of assumed clusters was set to a range going from 1 to 20, but each step was repeated 5 times. The best K was estimated with Structure Harvester^71^, then Structure software was run again for the best K, 50 times. Clumpak on the web^74^ was used to summarize the best K posterior probabilities and to graphically represent the bar plot output average over the 50 repetitions.

Matrix of the pairwise F_ST_ genetic distances between populations of each species, together with Bonferroni corrected *p* value, were achieved using Genepop v4.7^67^. Correlation between genetic differentiation and geographic distances between sampling locations were tested (either F_ST_ vs the combined distances along the minimum spanning tree between locations or F_ST_/(1 − F_ST_) vs log-transformed Euclidean distances between locations), in order to detect isolation by distance (IBD)^75^, using the ISOLDE program in Genepop^67^.

### *Kdr* mutation identification

#### PCR–RFLP visual reading

A polymerase chain reaction–restriction fragment length polymorphism (PCR‐RFLP) diagnostic assay developed by Tsagkarakou, et al*.*^41^ was used in this study to detect the mutation known to confer resistance to pyrethroids (i.e., the resistant *kdr*). Two mutations on a portion of the voltage-gated sodium channel gene are responsible for this resistance for the MED species (linked to two described non-synonymous mutations, either of them conferring an amino acid change: L925I and T929V), and one for the MEAM1 (involving the first locus and amino acid change: L925I). This diagnostic assay was performed on the whole dataset. Briefly, a 184 bp fragment was amplified (see Supplementary Table S3 online) following the PCR amplification protocol described by Tsagkarakou, et al*.*^41^—with slight modifications—and using GoTaq^®^ DNA Polymerase provided by Promega™. The PCR amplified fragment was then fully digested with restriction enzyme DdeI (Promega™), yielding fragments of different size depending on the *kdr* genotype. As susceptible allele contains one site for restriction enzyme DdeI, digestion of the PCR product results in a restriction pattern of two fragments (124 and 60 pb). The restriction site is non-functional when the resistant allele is present, leading to an intact fragment of 184 pb after enzyme digestion. This method allows to visualize homozygous such as heterozygous individuals at this locus. The QIAxcel^®^ Advanced Instrument, an automated capillary electrophoresis device supplied by Qiagen™, was used to perform gel electrophoresis and to visualize restriction patterns.

#### *Kdr* mutation sequencing

The sodium channel *kdr* region was sequenced for 12% of the whole dataset (including: 98% of all MED, 7% of all MEAM1, 7% of all IO, and 100% of hybrid genotypes) using the above-described primers and methodology. Then, PCR products were sequenced in both directions by Macrogen Europe laboratory (see Supplementary Table S3 online). Sequences were manually edited and aligned using Geneious™ software R10.2.6^64^. The resulting consensus sequences were compared with reference sequences from GenBank using the BLAST algorithm.

### Interspecific hybridization

Interspecific hybridization between the different species was assessed using the Bayesian clustering analysis performed by structure software, and further confirmed by the DAPC analysis. Individuals were considered hybrid when their assignment to the MEAM1 or IO cluster was comprised between 10 and 90%^39,76^. We used Kruskal–Wallis one-way analysis of variance (i) to assess the effect of the environment (greenhouse, open field, field surroundings or non-cultivated area) on assignment probabilities of hybrids (i.e., direction of backcrosses), and (ii) to test if there was a link between the *kdr* genotype and assignment probability to parental species.

## Supplementary Information

Supplementary Information.

Supplementary Table S1.

Supplementary Table S2.

## Data Availability

The datasets generated during and/or analysed during the current study are available in the zenodo repository at 10.5281/zenodo.5565437 and at https://dataverse.cirad.fr/.
