# Supplementary material for: Distribution of invasive versus native whitefly species and their pyrethroid knock-down resistance allele in a context of interspecific hybridization
Source: Sci Rep. 2022 May 19;12:8448. doi: 10.1038/s41598-022-12373-4 (PMC9120063; doi:10.1038/s41598-022-12373-4)
Supplement: Supplementary file 1 — Supplementary Information. [file 41598_2022_12373_MOESM1_ESM.pdf]

DISTRIBUTION OF INVASIVE *VERSUS* NATIVE WHITEFLY SPECIES AND THEIR PYRETHROID KNOCK-DOWN RESISTANCE ALLELE IN A CONTEXT OF INTERSPECIFIC HYBRIDIZATION

Alizée Taquet,[1,4] Hélène Jourdan-Pineau,[2,3,4] Christophe Simiand,[4] Martial Grondin,[4] Benoit Barrès,[5†] and Hélène Delatte [6,7*†]

[1] Université de La Réunion, Département de biologie, Campus du Moufia, 15 avenue René Cassin, CS 92003, 97744 Saint-Denis Cedex 9, La Réunion, France.

[2] CIRAD, UMR ASTRE, Campus international de Baillarguet, 34398 Montpellier Cedex 5, France.

[3] ASTRE, Univ Montpellier, CIRAD, INRA, Montpellier, France.

[4] CIRAD, UMR PVBMT, Pôle de Protection des Plantes, 7 chemin de l'IRAT, 97410 Saint-Pierre, La Réunion, France.

[5] Université de Lyon, ANSES, INRA, USC CASPER, Lyon, France.

[6] CIRAD, UMR PVBMT, Ambatobe, 101, Antananarivo, Madagascar.

[7] FOFIFA CENRADERU-DRA, Ambatobe, 101 Antananarivo, Madagascar.

[†] Equal contribution

* Correspondence and requests for materials should be addressed to H.D. (email: helene.delatte@cirad.fr)

Co-authors: alizee.taquet@univ-reunion.fr, benoit.barres@anses.fr, helene.jourdan@cirad.fr, christophe.simiand@cirad.fr, martial.grondin@cirad.fr.

Supplementary information

Supplementary Table S1 (see file attached): F_{ST} matrix for MEAM1 populations. All bolded numbers are significant (Bonferroni corrected *p*-value). Populations referenced by the number of the site (as referred to in Table 1) and environment: FS for field surroundings, G for greenhouse and OF for open-field.

Supplementary Table S2 (see file attached): F_{ST} matrix for IO populations. All bolded numbers are significant (Bonferroni corrected *p*-value). Populations referenced by the number of the site (as referred to in Table 1) and environment: FS for field surroundings, G for greenhouse, NC for non-cultivated area and OF for open-field.

Supplementary Table S3: Loci used for nuclear and mitochondrial DNA analysis (LN: locus name), source reference and primer sequence. This supplementary table was modified from Tsagkarakou, et al.[1] and Ally, et al.[2].

LN	Reference	Primer sequence (5'-3')
MS145	Dalmon, et al.[3]	F: CCTACCCATGAGAGCGGTAA
		R: TCAACAAACGCGTTCTTCAC
P59	Delatte, et al.[4]	R: TTTGCCAACTGAAGCACATCAATCA
P7	Delatte, et al.[4]	F: AGGGTGTCAGGTCAGGTAGC
		R: TTTGCGTAATAGAAAA
WF2H06	Hadjistylli, et al.[5]	F: TATTCGCCAATCGATTCCTT
		R: CGGCGGAAATTTCGATAAA
WF1G03	Hadjistylli, et al.[5]	F: CTCCAAAATGGGACTTGAAC
		R: GTAGAAGCCACACATACTAGCAC
WF1D04	Hadjistylli, et al.[5]	F: GTTGTTAGGTTACAGGGTTTGTC
		R: GTCTTTACTTCTTTTCCTCCG
P5	Delatte, et al.[4]	F: ATTAGCCTTGCTTGGGTCCT
		R: TTTGCAAAAACAAAAGCATGTGTCAAA
CIRSSA2	Ally, et al.[2]	F: ACAATGCATGTTGATTGTGAA
		R: TGAAAATGTCTACGGCCAGA
CIRSSA6	Ally, et al.[2]	F: CATATCGGTCATTATCCGCA
		R: CATCAGGCTGGAAAGACGAG
CIRSSA13	Ally, et al.[2]	F: AGTGCTGAAGGTCCACCGTA
		R: GGGATTTCCAGGGGTTAAGA
CIRSSA41	Ally, et al.[2]	F: TGGGTGCATGGTTCTTACAG
		R: TATCCGGTCGACAAACACAA
IIS4-5	Morin, et al.[6]	F: GCCAAATCCTGGCCAACT
	Tsagkarakou, et al.[1]	R: GAGACAAAAGTCCTGTAGC
mtCOI	Mugerwa, et al.[7]	F: TGRTTTTTTGGTCATCCRGAAGT
		R: TTTACTGCACTTTCTGCC

Supplementary Figure S1: Backcross direction (as indicated by the probability of assignment to MEAM1) of the 29 identified hybrids, in relationship to sampled environment.

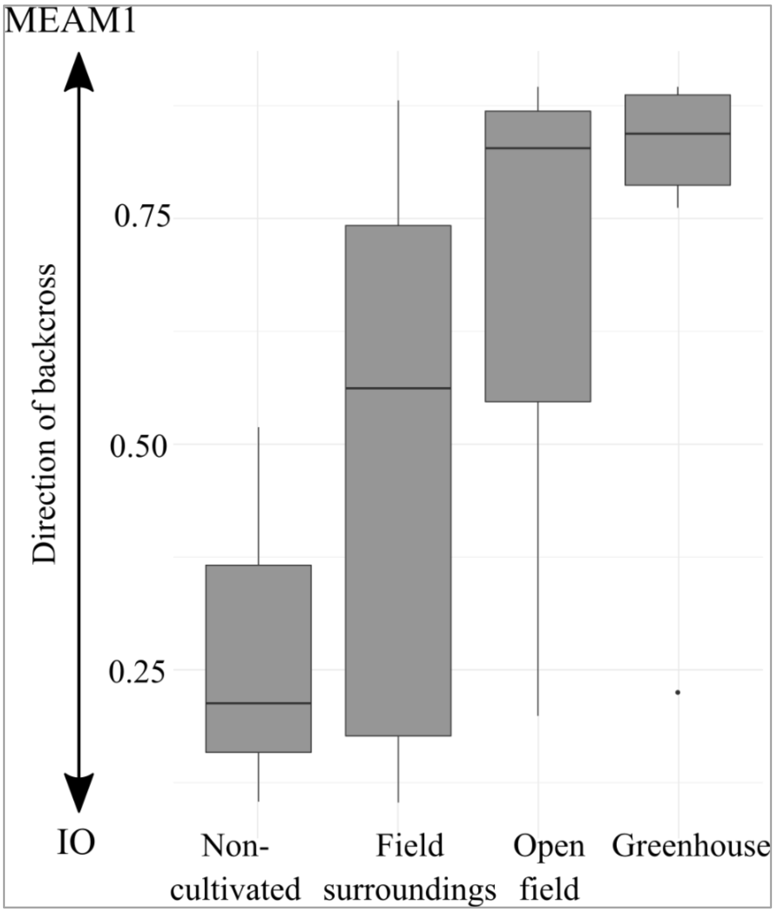

References

1 Tsagkarakou, A. *et al.* Molecular diagnostics for detecting pyrethroid and organophosphate resistance mutations in the Q biotype of the whitefly *Bemisia tabaci* (Hemiptera: Aleyrodidae). *Pestic. Biochem. Phys.* **94**, 49-54 (2009).

2 Ally, H. M. *et al.* What has changed in the outbreaking populations of the severe crop pest whitefly species in cassava in two decades? *Sci. Rep.* **9**, 1-13 (2019).

3 Dalmon, A., Halkett, F., Granier, M., Delatte, H. & Peterschmitt, M. Genetic structure of the invasive pest *Bemisia tabaci*: evidence of limited but persistent genetic differentiation in glasshouse populations. *Heredity (Edinb)* **100**, 316-325 (2008).

4 Delatte, H. *et al.* Microsatellites reveal extensive geographical, ecological and genetic contacts between invasive and indigenous whitefly biotypes in an insular environment. *Genet. Res.* **87**, 109-124 (2006).

5 Hadjistylli, M., Schwartz, S. A., Brown, J. K. & Roderick, G. K. Isolation and characterization of nine microsatellite loci from *Bemisia tabaci* (Hemiptera: Aleyrodidae) Biotype B. *J Insect Sci* **14**, 148 (2014).

6 Morin, S. *et al.* Mutations in the *Bemisia tabaci* para sodium channel gene associated with resistance to a pyrethroid plus organophosphate mixture. *Insect Biochem. Molec.* **32**, 1781-1791 (2002).

7 Mugerwa, H. *et al.* African ancestry of New World, *Bemisia tabaci*-whitefly species. *Sci. Rep.* **8**, 2734 (2018).
